# The Relationship between Exposome and Microbiome

**DOI:** 10.3390/microorganisms12071386

**Published:** 2024-07-08

**Authors:** Giuseppe Merra, Paola Gualtieri, Giada La Placa, Giulia Frank, David Della Morte, Antonino De Lorenzo, Laura Di Renzo

**Affiliations:** 1 Department of Biomedicine and Prevention, Section of Clinical Nutrition and Nutrigenomics, University of Rome Tor Vergata, 00133 Rome, Italy; 2 Ph.D. School of Applied Medical-Surgical-Sciences, Univeristy of Rome Tor Vergata, 00133 Rome, Italygiulia.frank@ymail.com (G.F.)

**Keywords:** microbiome, exposome, omics, commensals, organisms, environmental

## Abstract

Currently, exposome studies include a raft of different monitoring tools, including remote sensors, smartphones, omics analyses, distributed lag models, etc. The similarity in structure between the exposome and the microbiota plus their functions led us to pose three pertinent questions from this viewpoint, looking at the actual relationship between the exposome and the microbiota. In terms of the exposome, a bistable equilibrium between health and disease depends on constantly dealing with an ever-changing totality of exposures that together shape an individual from conception to death. Regarding scientific knowledge, the exposome is still lagging in certain areas, like the importance of microorganisms in the equation. The human microbiome is defined as an aggregate assemblage of gut commensals that are hosted by our surfaces related to the external environment. Commensals’ resistance to a variety of environmental exposures, such as antibiotic administration, confirms that a layer of these organisms is protected within the host. The exposome is a conceptual framework defined as the environmental component of the science-inspired systems ideology that shifts from a specificity-based medical approach to reasoning in terms of complexity. A parallel concept in population health research and precision public health is the human flourishing index, which aims to account for the numerous environmental factors that affect individual and population well-being beyond ambient pollution.

## 1. Introduction

Synthetic biology approaches are multiplying the number of bacteria that can be probed to a nearly complete repertoire of organisms that plausibly can be domesticated in the laboratory. This advancement is opening the door to unprecedented studies of the genetics and biochemistry that give rise to the correlations between the exposome and the microbiome. This is an understudied area, considering the extent of ecological variability in human lived environments and personal behaviors. Existing precision health strategies routinely ignore or marginally include general microbial aspects of the exposome. The main reasons for this gap are the increasing list of microbial–host correlations that seem to be specific for members of the microbiome and its variation in environmental, demographic, and pathophysiological contexts. However, we argue that growing availability of metadata like general and specific reference sequences, and environmental and social surveys, together with the pioneering studies on synthetic microbiology, are opening affordable avenues for the domestication and genetic manipulation of a vast quantity of environmental bacteria not yet cultivated in the laboratory and many that still are unculturable [1,2]. The microbiome and the exposome, two recent scientific concepts, have aroused considerable interest, particularly within the context of health research. The microbiome, defined as all bacteria, archaea, viruses, and eukaryotic organisms within and on the human body, has been the focus of intense research activity during the last decade. In the same period, the exposome concept emerged as an attempt to encompass all exposures from the external environment and any biological endogenous system exposures, including those internal to the body. The focus of this article will be to review recent evidence of microbial aspects of the exposome and exposome aspects of the microbiome.

### Definition and Scope of Exposome and Microbiome

The human microbiome consists of the microbial community (including bacteria, archaea, fungi, viruses, and microfauna) connected with the human body; more than 90% of microorganisms in the healthy human body live in the intestine [3]. It forms a dynamic ecosystem that is not completely shared by all humans. There is a so-called “core” microbiome, but its extent remains a matter of discussion, and diet, antibiotic use, and other causes can modify the composition of the intestinal microbiome. The majority of the 75% of this microbiome that is composed of bacteria are classified as Firmicutes, Bacteroidetes, Actinobacteria, Proteobacteria, and Fusobacteria. The microbiota supplies the host human body with several essential products such as vitamins, influences the immune system by developing specific types of lymphocytes, and teaches the oral and gulate epithelia how to differentiate (in other words, it plays a significant role in pharmacology and effectively degrades and absorbs some of the drugs). To discover the specific details of microbiota activity, it is necessary to study both stable and dynamic changes in microbial communities by associating these with microbiota genetic changes. The term “exposome” was coined by Christopher P. Wild in 2005 and, contrary to the notion of the genome, encompasses all human environmental exposures from conception onwards [4]. It introduces a holistic approach to health, considering social factors, chemical and radiotoxic agents, and physical influences such as ultraviolet radiation, temperature, noise, and climate. The exposome consists of three domains: (i) the general external environment, including air, water, soil, food, climate, habitat, and infrastructures; (ii) the specific external environment, including technospheric external products, drugs and cosmetics, professional activity-related pollutants, all sorts of dietary products, and supplemented chemicals; and (iii) the internal environment, including genetic properties, health status, health behaviors, psychosocial and economic environments, and individual microbiota. Both humans and mesoorganisms can be incorporated into the exposome, as the human exposome is exposed to a microbiota and a metacommunity, while the microbiome of mesoorganisms (e.g., the human gut microbiome, the gut mycobiome, and the urian microbiome) is exposed to the human exposome. The microbiome is now included in exposome research despite not being a part of the original proposal and can also be integrated into the exposome. As a result, we have three levels of biological organization involved in exposomics, including individual exposomics (in human biology), specific mesoexposomics (i.e., the microbiome exposome), and general mesoexposomics (e.g., animal and plant ecology applying metacommunity theory). Thus, the exposome concept aims to provide a comprehensive environmental exposure history in order to better understand its impact on human health and well-being and its interplay with human genomics.

## 2. Understanding the Exposome

We aimed to conceptualize the occupational exposome as a network of occupational health factors (OHPs) shared with common occupational exposure followed by constructing an integrated framework. To express collective relationships in this occupational exposome, we used an adjacent matrix, where components are OHPs with indivisible influence and zero interference. To conceptualize the construction of an integrated network that links heterotypic occupational health factors, systemic environmental exposure networking, and individual differences, we propose introducing the term “occupational exposomics”. Then, we define it as the construction and analysis of the whole and dynamic network that the occupational exposome, host adaptome, and human microbiome inhabit. It is also used to identify the OHPs that carry social implications from a network based on the same method, but information on occupational exposure unrelated to the human body, such as changes in workplaces or generation materials resulting from activities yielding production, is used. In addition, occupational and social association networks are derived based on the analysis of adjacency matrix values, indicating that there was a basis for some similar OHPs, but further confirmation is necessary to determine the robustness of social OHP clustering. In 1998, the World Health Organization highlighted the potential synergy between environmental and social exposure; when these exposures often occur within an occupation, it is referred to as the occupational environment [5]. However, due to the poor visibility of various types of occupational health indicators (OHPs) and the lack of comprehensive exposure assessments [6], and though academic efforts have been made to organize and understand these health factors, it is still difficult to identify the potential hazards of the occupational environment. Since 2005, the goal of the examination of systemic environmental influence on human health and the realization of a holistic approach has been emphasized in various academic fields, including psychology, environmental science, and public health. Furthermore, the human microbiota and organisms have been focused on as important factors that mediate the influence of the exposome on health [1]. Therefore, the construction of an integrated network that links occupational health factors, social factors, microbial flora, and the host genome, and that incorporates advanced statistical models, is necessary to study the association between environmental and social exposure in the occupational environment and host health outcomes.

### Conceptual Framework and Components

Direct interactions between the microbiome and environmental exposures are a key focus, as is the integration of host and microbiome-related omics, which stresses the complexity of this interaction. Among the exposomic components that were studied for their interaction with the microbiome, life-course studies revealed great promise. This perspective outlined the potential of combining omics-based exposures—including metagenomics, which contributes to the exposome definition in the host—and epigenomics-based omics, together with the exposome concept [7]. This move seeks to reflect the development of the exposome concept from an external “utilitarian” view to an internal view with an emphasis on biological responses at molecular and cellular levels. The intriguing prospect of investigating personalized recombinant environmental risk factors associated with the responses over the course of life should become explicable unless the molecular and biological levels are added. Researchers from various scientific fields are trying to shed light on the mechanism of how complex environmental exposures shape human traits. Whereas both the exposome [1] and the microbiome have been studied separately as means of identifying novel sources of disease risk and discerning mechanisms, it has increasingly come into focus in the last few years to consider both concepts as highly interconnected. It has been demonstrated that the exposome can shape the (host-associated) microbiome, not only giving this innovative research—being an exposure-driven microbial ecosystem—great promise for personalized health applications but also bearing the potential for accelerated discovery and potential intervention for prevalent diseases, including infectious diseases, chronic diseases, and cancer. The sheer size of the datasets that necessitate integration in order to understand the exposome and microbiome nexus requires advance bioinformatic methods to remedy the remaining technical and statistical pitfalls of the revealed entities from associations.

## 3. Understanding the Microbiome

The human microbiome is a vast and complex assemblage of over a thousand microbial species and has been the focus of exceptional attention due to its apparently significant roles in human health [3]. Its composition and functioning need to be taken into account not merely at the whole organism level but also in the individual bodily compartments, which together constitute an ecosystem. Notwithstanding, considering only microbially driven metabolic processes could fail to unveil the whole molecular bases of host–microbiome mutualism, which also comprises communication through enzymes, secreted peptides (in some cases in reciprocal regulation), and toxins [8]. This network of reciprocal regulations appears to be functionally integrated through epigenetic mechanisms. The mutual interaction between the body’s cells and the resident bacteria has a substantial impact on various aspects of the host architecture, including the immune system, immediate metabolism, hormonal balance, the brain, and development [9]. The host response to bacterial presence has to be adapted to the peculiar circumstance of each microcompartment, where the pressure of the microenvironment or the physiological/growth state of the eukaryotic host might be substantially different. This individual adaption to the bacterial presence necessitates the respect of the very specific personal expression program.

### Definition and Importance

We can suffer progressive damage to genetic information, proteins, lipid bilayers, lipoproteins, and carbohydrates, and a chronic inflammation–oxidative stress synergism that affects the kinasome. We can divide the comparome into proactive and reactive sections, subject to progressive damage. Microorganisms, that is, the human microbiome and its genes, can adapt or evolve to these alterations in cellular physiology and pathological processes. The human microbiome is more than exposure, concentration, and time: It is an exogenous inducer that interacts in the human phenotype [10]. However, the microbiome is now included in exposome research despite not being a part of the original proposal. As defined in a commentary by Walsh, the microbiome can also be integrated into the exposome. Various studies have already suggested that the microbiome greatly affects the human health system, which is why microbiome research is just as important as exposome research. The relevance of the exposome–phenotype relationship is so important that some researchers suggest using the relatome concept as a methodological framework to understand the influenced microbiome–exposome relationship [5]. Researchers in the field of microbiome research have proposed a unified concept—“the exposome and microbiome in health and disease”—or, simply, “the relatome” [3], to understand bi-directional interactions. The focus of exposome studies is on non-genetic environmental factors, including human lifestyle, diet, chemicals, ionizing radiation, UV radiation, microorganisms, drugs, and stress. The microbiome, on the other hand, mainly focuses on ecosystems, including bacteria, viruses, fungi, protists, and archaea, and investigates the direction and strength of interactions between the microbiome and the environment. Although the exposome and the microbiome are independent of each other, the relationship between the two is strong.

## 4. Interactions between Exposome and Microbiome

Genes, your genetic heritage, are the first domino in the pathogenesis of most human diseases—but in most cases they are not sufficient to drive the disease. Genes interact with many environmental factors and are kept under strict control by the epigenome, which responds acutely to the exposome. The exposome, then, always acts “downstream” on genetics and epigenetics, and the “black box” that contains many of the biologically and, thus, diagnostically and therapeutically relevant sensing pathways probably also depends on the activities of the gut microbiota and their “metabolome effects”, facilitating the potential of the microbiome and the exposome as a broader angle of view. These two innovation granitic tools will be instrumental in fostering a personalized preventive and predictive medicine that could drastically reduce the proportion of “end of the pipe” diagnostic procedures and personalized pharmacological tools, with obvious benefits for the sustainability of national health systems around the world [11]. The exposome and the microbiome are two emerging concepts that offer crucial new insights into human health and disease. The exposome represents the totality of exposures (both external and internal) over our lifetimes, expressing the dynamic interactions between the microbiome, the air, our diet, the physical environment (buildings, neighborhoods, cities, and nature), the products we use, and the lifestyles we follow. The exposome influences immune homeostasis as well as the neuroimmune system and responds acutely to external factors, thus mirroring the human responses to socioeconomy, built environment, habits, behaviors, culture, and climate change, and giving rise to environmental exposures, endocrine and environmental disruptors, xenobiotics and antibiotics, food and air allergens, and viruses and bacteria. Notably, many of the exposome factors can modulate the microbiome (air, water, soil, and, first and foremost, the gut microbiome) in a matter of days/weeks and can generate microbial metabolic bioactive compounds that have profound biological effects on various target organs. The interactions between the different components of the exposome in shaping the microbiome and the relative metabolome can impact, for example, the risk and exacerbation of severe allergic asthma.

### Direct and Indirect Interactions

Here, we will review some hypothetical direct (mechanistic) and indirect (i.e., not necessarily tight cause–effect links) interactions between the exposome and metagenomic measures of the gut microbiota [12]. First, the gut microbiota could contribute to or directly drive metabolism and immune function through the metabolism of xenobiotics (external exposome), energy from the host diet, and key nutrients, and/or through the production of metabolites such as vitamins, short-chain fatty acids, and bile acid derivatives. Second, the gut microbiota could be directly modulated through the environmental influx of different xenobiotics (e.g., drugs and food additives). Third, alterations in gut microbiota membership and function could indirectly reflect/shape over time differential exposure to environmental deleterious agents—for instance, body enlargement with aging (e.g., from lifestyle changes such as decreased physical activity and exposure to energy-dense foods, after grants to prolonged lifespan whose potential feedlot can be overridden by exposure to xenobiotics from food and drugs), or inflammation due to exposure to environmental perturbations such as particulate matter air pollution over the very long lifetime of an organism. A rich understanding of the relationships between the environment and the microbiome will need to account for complex direct and indirect interactions between these two systems that are shaped by functional processes of the host and molecular composition characteristics of environmental exposure [11]. From an exposomic point of view, while the concept had mainly been proposed to integrate the external and non-genetic internal determinants of human physiology, the gut microbiota and its bacterial metagenome, namely, the intestinal metagenome, fall squarely within the exposome’s sphere, making the exposome and the gut microbiota close relatives within a single system where exposures, measurements of biological perturbations, and phenotypic traits such as susceptibility to diseases can be seen as different sides of the same coin. While the exposome was initially defined simply as the totality of all external exposures and other perturbations to which an organism is subjected from conception until death, and their response to these perturbations summed over the entire lifespan of the organism, it is now considered that an organism’s internal exposome is created in addition to (or sometimes in response to) its external exposome and constitutes everything within its physiological borders. Namely, the observations at each exposure time of its metabolome, its entire repertoire of proteins and transcripts, its ion abundance, its electrophysiology, and its singe-cell and non-cell autonomous interactions are often referred to as the internal exposome.

## 5. Methods for Studying Exposome–Microbiome Interactions

With recent advances in analytical chemistry and omics technologies, exposome characterization has become feasible. Determinants and mechanisms of action between the exposome and the microbiome can be analyzed to elucidate their interaction by using several candidate approaches [13]. Different combinations of physiological biomarkers and multi-omics techniques can be integrated into different exposure bio-monitoring approaches. Use of multi-omics techniques with multi-center health studies will improve exposome and personal exposome exposure data and can be used to identify informative metabolic biomarkers, especially for more complex models. Multi-omics techniques can be linked with results of exposure monitoring to improve understanding of the biological response to environmental exposures. Uptake of exposure monitoring and carry-over meta-component profiling can be used to identify novel sources and pathways of environmental exposures correlated with exposure monitoring data at the population level, such as regulatory exposure biomarkers, and regulatory exposure biomarkers and meta-component profiling can be used to improve mechanistic understanding in the human health-risk assessment process. Exposures in the environment exert multiple mechanisms of action on the human host. Microbial colonization begins at birth and bacteria colonizing the human host play crucial roles in the immune system and have a profound effect on the gut–brain axis via gut microbiota-related metabotypes, which are used to set microbial predictors [14]. In particular, the reciprocal relationship between the environment and the human microbiome is reflected in the bidirectional interaction between the exposome and the microbiome. The exposome was first detailed as a measure of environmental exposures accumulated over the human lifetime. The exposome is divided into the general environment, including exogenous factors, and the internal environment, including endogenous factors. The microbiome is considered one of the internal environmental factors.

### Omics Technologies

In a sense, the composed internal phenome that measures inherited genetic, acquired somatic and tumor genetic, serum cytokine and autoantibody (and other agent) responses, and circulating cell-free DNA, in addition to the microbiome and above-mentioned biomes and the external exposome that potentially impact these exposures and actions that act in concert on human health and disease, are two super-integrated and synergistic systems in the original exposome paradigm [15]. The uninterrupted and intercurrent analytics and zeitgeber of the former are the ensemble of tightly regulated, sequenced, organized, chance-determined, or even simultaneous pathophysiological circuits layered over the basic ordered hardware of biological processes and systems, building up efficient circuits replete with regenerative potential, etc., but with cracks, avoided regions, and dead membranes as well. For the practitioner, it is action, knowledge, information, and knowledge of (e.g., understanding) action. The original exposome has evolved significantly, and the concept now extends to chemicals, particulate matter, air pollution, infections, toxin exposures, diet, lifestyle, hobbies, and behavior [16]. Each of these agents has an omics signature that includes many diverse molecular components and potential molecular effectors, and they may contribute to different health effects variously depending on the exposure pathway and dose, the population expression of metabolic enzymes and other bioactivating or detoxifying agents, and the background of other risks, especially those that are communicable, that can modify host gene expression (epigenome) and liability to predisposing or exacerbating chronic disease and immune-related outcomes [17]. For example, altered flux of monosaccharides that are extracted with the food, modified with bioactive metabolites by the intestinal microbiota, and diffused across the human epithelia can result in an altered post-prandial metabolic state. Dysbiosis, not just at the level of the microbiome but also at the level of other biomes, such as the virome, mycobiome, and bile acid pool; alcohol dehydrogenase expression in the oral cavity; and butyrate fermentation, has proven to have different effects on health.

## 6. Health Implications of Exposome–Microbiome Interactions

The gut microbial community not only actively functions to degrade various metabolites but also plays an inseparable role in affecting host biological behaviors (including genetics and physiology) via an internal metabolic network. This presents potential opportunities to optimize developmental outcomes among young children. By taking a more holistic and preventive approach to early life, we could develop new, personalized strategies to underpin optimal child health outcomes, which goes beyond simply manipulating the infant gut microbiota to consider more broadly the child’s entire lifetime exposure history and how these could feed upwards into their health profile through complex gene-by-exposome-by-microbiota interactions. Understanding the interplay of the exposome and the microbiome within the gut is needed to understand how these external exposures may predispose an individual to a certain endophenotype and, as such, has real potential to enhance current practices in the provision of healthcare [18]. The exposome provides a new perspective on how environmental exposures may work synergistically with human genetics and physiology to regulate biology and influence health [19]. It is important to acknowledge that the complicated interactions between environmental exposures and health frequently occur across multiple molecular and cellular levels and are often shaped, in part, by the gut microbiome. Therefore, understanding the connections between the exposome and the microbiome has the potential to substantially augment our understanding of how non-genetic factors contribute to adverse health outcomes. The composition and diversity of the gut microbiome is influenced by environmental chemical exposure, and research has demonstrated the role of the gut microbiome in the metabolism of xenobiotics from the chemical exposome.

### Impact on Disease Susceptibility

While differences in the composition of the microbiome and its impact on the development of the nervous system and associated behavior, particularly when talking about mice and the gut–brain axis, were already observed years ago, it was only in the last decade that researchers on humans started to unravel the importance of the microbiome for disease onset and progression—for example, for obesity, various cancers, diabetes, and many others. The observation that mice lacking gut microbial content have altered stress responses had been made earlier. Later studies showed that adult animals raised germ-free exhibit lower levels of anxiety-related behaviors compared with animals specifically maintained under conventional conditions. This implies a crucial role of the microbiome in the maturation of the HPA axis and regulation of the host stress responses. It has recently been demonstrated that the bidirectional communication of the gut–brain axis had probably started immediately after birth, with the development of the microbiome influencing the trajectory of neurocognitive development by impacting stress regulation; immune, metabolic, endocrine, and neurotransmitter systems; and synaptic and myelin plasticity. Individuals with higher overall microbial diversity, including alpha and beta diversity, are generally healthier at baseline and may be better protected from chronic diseases [20]. On the other hand, a variety of environmental exposures are linked to an increased risk of developing many chronic diseases. Mental disorders, often associated with chronic diseases, can potentially be initiated or exacerbated by various behavioral and social factors, lifestyle changes, and stressful life events. These mental health issues have also been connected in various ways with the exposome, such as in our recent scoping review on the exposome and mental health, where external factors such as air pollution, noise, and negative life events were among the factors associated with mental disorders [21].

## 7. Therapeutic Applications and Future Directions

Currently, therapeutic strategies are often based on one or more isolated factors, such as the administration of a certain vaccine, dietary supplement, or medication. In many cases, even the combined effects of multiple treatments remain poorly understood. A modular conception of the individual highlighted the need to explore larger scientific models, i.e., biological systems that operate within complex relationships and exchanges in their environment. In this context, the exposome–microbiome intercommunication appears decisive, in terms of both the prevention and the treatment of multifactorial human diseases. In the context of therapeutic intervention, prebiotic and probiotic strategies appear relevant to increasing the efficacy of nutritional, pharmacological, and environmental interventions. The five main strategies currently being investigated are transfer/nutritional modulation of the microbiome, direct/indirect modulation of the exposome, protective intervention with natural ingredients, reprogrammed metabolite export, and barrier structure reinforcement [22,23,24]. Dysbiosis of the gut microbiome has been linked to a variety of health issues, including cancer and infectious, allergic, metabolic, and neurological diseases. The vast literature of epidemiological studies shows that exposure to a plethora of specific chemical and physical agents is unequivocally associated with an increased risk of developing several types of cancer. We will review this evidence, stressing its relevance for the concept of the exposome, which strives to measure all relevant environmental risk factors acting at the individual and population level. We will critically assess the magnitude and strength of the epidemiological association, the biological plausibility of the underlying mechanisms, and to what extent longitudinal monitoring of the exposome may add to our understanding of the health appraisal and risk threshold values set by regulatory agencies. The role of the environment in driving the development of cancer is increasingly well documented. Mounting human and experimental data show the impact of individual environmental pollutants on important cellular processes that, when deregulated, can lead to the development of cancer. In this chapter, we discuss the evidence linking different environmental exposures to the development of cancer and provide an exposome-based view of cancer causation. The concept of the exposome encompasses populations, demographics, habits, and lifestyles, as well as the physical, chemical, and biological characteristics of the environment, including all conditions that influence risks throughout life [25,26].

For this reason, microbiome-related modulations involving food or suppository supplementation with defined bacteria or bacterial products may have important therapeutic implications. During the transfer of the microbiome from healthy to poorly defecating mice, rectal irrigation with human fecal samples in disease-free offspring was shown to relieve the symptoms of Parkinson’s disease. The value of influencing the microbiome is also being increasingly recognized in cancer research. A meta-analysis of 10 original controlled non-human treatment studies (of which 2 were randomized) recommends fecal transplant and probiotic treatment as a potential cancer therapy [23,24].

### Probiotics and Precision Medicine

Commercial usage of probiotics, prebiotics, and postbiotics is increasing. Therefore, it appears that we are entering an era of “live bacteria as drugs”. Refinement of predefined probiotic strains, or the development of tailored user-specific probiotics, prebiotics, and postbiotics, is in this respect promising for achieving personalized health protection. In order to retrieve strong links between the microbiome and clinical disease, a better understanding is needed of how gut microbes can specifically shape a host’s immune responses. It is suggested that the application of systems biology approaches to human gut models in combination with current human metagenomic-linked genetic immunology studies, as well as ongoing gut bacterial and host immunology studies, will not only provide the missing links but also set the stage for improved preventative microbiome therapeutics. Longitudinal comprehensive projects for healthy and diseased individuals across human life courses in that respect are needed. The concept of “precision medicine” or “personalized medicine” is gaining momentum, as it has been shown that a patient’s health can be significantly shaped by its individual microbiome, genetics, and environmental factors, such as diet, physical activity, and living patterns [23]. Probiotics, by definition, are live microorganisms that can improve the health of the host if administered in adequate amounts. Current therapeutic applications of probiotics span many different fields, ranging from regulation of the endocrine system to metabolic homeostasis, and targeting of the cardiovascular, nervous, immune, mucosal, bone, and urinary systems [24]. Therefore, probiotics can be utilized as a tool for precision nutrition, especially for undernourished or obese individuals, by mimicking certain dietary practices. Moreover, bacterial or other microbial-based therapeutics have been employed to induce the desired health benefits. Microbiota-based therapies are not limited to probiotic use but also include the employment of prebiotics, symbiotics, postbiotics, single- or multiple-component extracts, microbiota transplantations, and fecal microbiota transplantations.
